# Isolation and Identification of Myxobacteria from Saline-Alkaline Soils in Xinjiang, China

**DOI:** 10.1371/journal.pone.0070466

**Published:** 2013-08-06

**Authors:** Xianjiao Zhang, Qing Yao, Zhuoping Cai, Xiaolin Xie, Honghui Zhu

**Affiliations:** 1 Xinjiang Production and Struction Corps Key Laboratory of Protection and Utilization of Biological Resources in Tarim Basin, College of Life Science, Tarim University, Alar, Xinjiang, China; 2 Guangdong Provincial Microbial Culture Collection and Application Key Laboratory, Guangdong Open Laboratory of Applied Microbiology, State Key Laboratory of Applied Microbiology (Ministry-Guangdong Province Jointly Breeding Base), South China, Guangdong Institute of Microbiology, Guangzhou, Guangdong, China; 3 South China Agricultural University, Guangzhou, Guangdong, China; Missouri University of Science and Technology, United States of America

## Abstract

Fifty-eight terrestrial and salt-tolerant myxobacteria were isolated from the saline-alkaline soils collected from Xinjiang, China. Based on the morphologies and the 16S rRNA gene sequences, these isolates were assigned into 6 genera, *Myxococcus*, *Cystobacter*, *Corallococcus*, *Sorangium*, *Nannocystis* and *Polyangium*. All the strains grew better with 1% NaCl than without NaCl. Some *Myxococcus* strains were able to grow at 2% NaCl concentration, suggesting that these strains may be particular type of terrestrial myxobacteria.

## Introduction

Myxobacteria are Gram-negative bacteria with rod-shaped vegetative cells that exhibit unique group behavior [1]. They can produce more than 100 types of secondary metabolites with unique structures and functions, and over 600 types of new analogues [2]. Thus myxobacteria have been regarded as “microbe factories” for active secondary metabolites, because they have great potential as a prolific source of new drugs for drug discovery programs [3].

Myxobacteria are widely distributed in natural ecosystem. However, currently less than 10% of all the natural species have been isolated [4]. Therefore, it would be of great potential economic value to improve our capacity to isolate new myxobacteria, in order to provide more strains for drug screening and development.

Myxobacteria are considered typical soil microbes [1], [5], [6], and no purified myxobacteria had been confirmed to be able to grow under salt concentration of more than 1.0% [1] until 1998. Previous study indicated that two swarm-forming bacterial strains had homologous 16S rDNA sequences (89.3% and 83.2% similarities) with *Nannocystis* [7], a lineage of myxobacteria [8], [9]. Some myxobacteria have been found to exist in marine environments as halotolerants or partial halophiles [7], [10]–[13]. For example, halotolerant and partially halophilic myxobacterial strains were isolated from a coastal area in Japan, resulting in the discovery of two new genera, *Plesiocystis* and *Enhygromyxa* [14], [15]. However, there have been no reports of myxobacteria isolated from terrestrial saline-alkaline environments. Here we report the isolation of 58 myxobacterial strains from saline-alkaline soils in Xinjiang, China. Some of these terrestrial myxobacterial strains could tolerate NaCl concentrations of 2%.

## Materials and Methods

### Soil Collection

Twenty-five samples of saline-alkaline soils were collected from Xinjiang, China. No specific permits were required for the described field studies. We state that no specific permissions were required for the locations where the soils were sampled and we confirm that the location is not privately-owned or protected in any way and that the field studies did not involve endangered or protected species. The samples were air-dried immediately after collection and stored at 4°C.

### Strain Isolation and Purification

The strains were isolated using conventional myxobacterial isolation methods. Living *Escherichia coli* was smeared in the form of a cross-streak on WCX (CaCl_2_.2H_2_O 0.1%; agar 1.5%; pH 7.2). After autoclaving, 25 µg/ml cycloheximide was added to the medium agar plate. 100 g soil was added to 1 L distilled water, boiled for 20 min, and then centrifuged to prepare the soil extracts. The central portions of the streaks were incubated with a pea-sized aliquot of the soil samples and moistened with sterile water. For the isolation of cellulose-degrading myxobacteria, slices of filter paper were placed on top of the ISCX agar (KNO_3_ 0.1%, FeCl_3_·6 H_2_O 0.02%, K_2_HPO_4_ 0.1%, MnSO4·7 H_2_O 0.01%, CaCl_2_·2 H_2_O 0.1%, MgSO4·7 H_2_O 0.1%, yeast extract 0.002%, agar 1.5%, pH 7.2). They were then inoculated in the center of the filter paper with a pea-sized aliquot of the soil samples. All plates were then incubated at 30°C. After three days, myxobacterial fruiting bodies began to be formed and were usually recognizable within 2–4 weeks. The fruiting bodies were picked daily and examined under a dissecting microscope. They were transferred to VY/2 medium (Barkers yeast 0.5%, CaCl_2_·2 H_2_O 0.1%, agar 1.5%, pH 7.2). For purification, the agar pieces from the fringes of the swarms were picked with needles and transferred to VY/2. The procedure was repeated until the swarms were pure. Rabbit dung pellet method was also used as described by Reichenbach [16].

### Systematic Identification

#### Morphological identification

All strains were incubated in VY/2 medium. Growth and morphogenesis were observed with dissecting microscope, optical microscope, and electronic microscope. The swarms were carefully scraped to glass slides for detection. Fruiting bodies were crashed to release myxospores for detection. According to *The Prokaryotes* [1] and *Bergey’s Manual of Systematic Bacteriology* [17], the taxonomy of the isolates were determined by the morphologies of vegetative cells, fruiting bodies, myxospores, and swarms?

#### 16S rDNA sequencing and phylogenetic analysis

Extraction of genomic DNA, PCR amplification and sequencing of the 16S rRNA gene were performed as described by Li *et al*. [18]. The PCR products were purified with UNIQ-5 column PCR products purification kit (Sangon) and sequenced at Yingjun Company. The 16S rRNA gene sequences of 58 strains were compared against a database of cultured species via BLAST analysis (http://blast.ncbi.nlm.nih.gov/Blast.cgi) and EzTaxon(www.eztaxon.org) in order to retrieve most similar sequences of recognized bacteria. All the sequences were deposited in GenBank, and the accession numbers were obtained (HQ623094-HQ623124, and K862589-KC862608). Multiple alignments were performed with the CLUSTAL_X software package [19]. The phylogenetic trees were constructed by the neighbour-joining [20] using the software packages MEGA version 4.0 [21].The topologies of the phylogenetic trees were evaluated by using the bootstrap resampling method of Felsenstein [22] with 1000 replicates.

#### G+C mol% assay

The G+C content of the genomic DNA was determined by using the HPLC method [23].

#### Fatty acid analyses

Strains were cultured on VY/2 agar for 5d at 28°C, and the fatty acid methyl esters were prepared according to the protocol of the Sherlock Microbial Identification System (MIDI system; http://www.midi-inc.com/) and analyzed by GC (6890; Hewlett Packard) using the microbial identification software package [24]. Analysis of whole cell hydrolysates was performed by GLC (Agilent 7890A) according to Kämpfer & Kroppenstedt [25] using the classical method of the Sherlock Microbial Identification System version 6.1 (MIDI) and the standard MIS library TSBA6. All the experiments were carried out in triplicate.

#### Multilocus Sequence Analysis (MLSA)

PCR primers for genes coding for *lepA* (encoding leader peptidase, a GTP binding membrane protein) and *gyrB* (encoding DNA gyrase, subunit B) were selected [26]. PCR products were sequenced at Yingjun Company. All the sequences were compared against a database of cultured species via BLAST analysis (http://blast.ncbi.nlm.nih.gov/Blast.cgi). The sequences were deposited in GenBank to get the accession numbers (KC914480-KC914531). The topologies of the phylogenetic trees were evaluated by using the bootstrap resampling method as previously described.

#### Effects of NaCl on growth

The effects of NaCl on growth were investigated on VY/2 agar media with 0–5% NaCl added. Each strain was inoculated onto the center of the agar plate and incubated at 30°C for 7–30 days. The growth of each myxobacterial strain was monitored by visual inspection.

## Results

### Collection of Soil Samples and Soil Properties

Most of the samples are sandy soils, and few are clays from saline-alkaline areas in Xinjiang, China. The vegetations are *Ligustrum lucidum, Phragmites australis, Chinese tamarisk, Suaeda giauca* and *Ziziphus zizyphus*, and half of the samples were collected from bare alkaline ditches around the areas. We measured the pH, organic matter content and salt content of soils. The pH ranged from 7.6 to 8.7, and the organic matter content was low, in the range of 5.9–32.6 g/kg. Most of the soil samples from bare alkaline ditches contained high salt content, with the highest one reaching to 346.8 g/kg (Table 1).

**Table 1:** The characteristics of 25 saline-alkaline soils.

Vegetation type	Sampling site	pH	Organic matter content (g/kg)	Salt content (g/kg)
*Ligustrum lucidum*	Nongyishiyituan	7.6	32.6	7.3
*Phragmites australis*	Nongyishishituan	8.4	15.2	31.7
	Shaya saline lake	8.4	9.6	67.9
	Shayabinghe load	7.8	14.9	70.8
	Kuche	8.1	8.4	69.9
*Chinese tamarisk*	Nongyishishituan	7.6	7.1	16.4
	Nongyishishituan	7.9	19.9	130.6
	Shaya er pasture	8.5	7.6	25.8
*Phragmites australis-Chinese tamarisk*	Nongyishishituan	8.1	22.7	6.6
	Ala-er TV station	7.9	11.0	46.5
	Xinhe	8.3	8.1	48.3
*Suaeda giauca*	Shayashisantuan	7.9	22.2	199.9
*Ziziphus zizyphus*	Nongyishishituan	8.5	6.1	34.7
Bare alkaline ditch	Shaya	7.9	11.3	85.1
	Nuerbage	8.6	6.3	79.9
	Bixibage	8.7	7.3	19.7
	Bozidun	7.9	10.8	76.3
	Akesu	8.6	11.1	132.8
	Nongyishisantuan	8.0	14.6	11.7
	Await	8.1	5.9	48.1
	Akesu	8.4	20.6	223.8
	Nongyishibatuan	8.4	30.4	346.8
	Liangfanchang	7.9	12.8	49.0
	Wushen	8.0	11.1	14.8
	Nongyishiwutuan	7.9	30.8	98.7

### Strain Isolation

Using rabbit dung pellets method, WCX method, filter paper method and soil extract method, 58 strains of myxobacteria from 25 soil samples were isolated from Xinjiang, China (Table 2). Rabbit dung pellets are natural medium for myxobacteria growth. Fruiting bodies can be induced in a shorter time (within 2–3 days) by sterilized rabbit dung pellets, and most of fruiting bodies are Myxococci that are easy to be purified. However, natural rabbit dung pellets are rich in organic matter, and meanwhile are easily contaminated by molds during the induction process, covering the fruiting bodies of myxobacteria. Among the 58 strains, only two were isolated by rabbit dung pellets method. Thirty-two strains were isolated by WCX method, and twenty strains by filter paper method, indicating these two conventional methods were able to isolate more myxobacteria species. Myxobacteria grew rapidly in the WCX medium, and they could be observed under optical microscope after culture for 3 days. While they grew slowly in the inorganic salt filter paper; they took 7–14 days or longer time to grow up. In order to imitate a soil environment to maintain the relationship among microbial populations, soil extracts were also added to be components of the growth medium, which resulted in an increased diversity of microbes. Nevertheless, this soil extract method brought about difficulties for purification, causing contaminants, and eventually only four strains were isolated by this method.

**Table 2:** Myxobacteria strains isolated by different methods.

Isolation methods	*Myxococcus*	*Corallococcus*	*Cystobacter*	*Sorangium*	*Nannocystis*	*Polyangium*	Total
Rabbit dung pellets method	2						2
WCX method	20	8	3			1	32
Filter paper method	8	5	3	2	2		20
Soil extract agar method	3	1					4

### Morphology Characteristics

**Figure 1:**
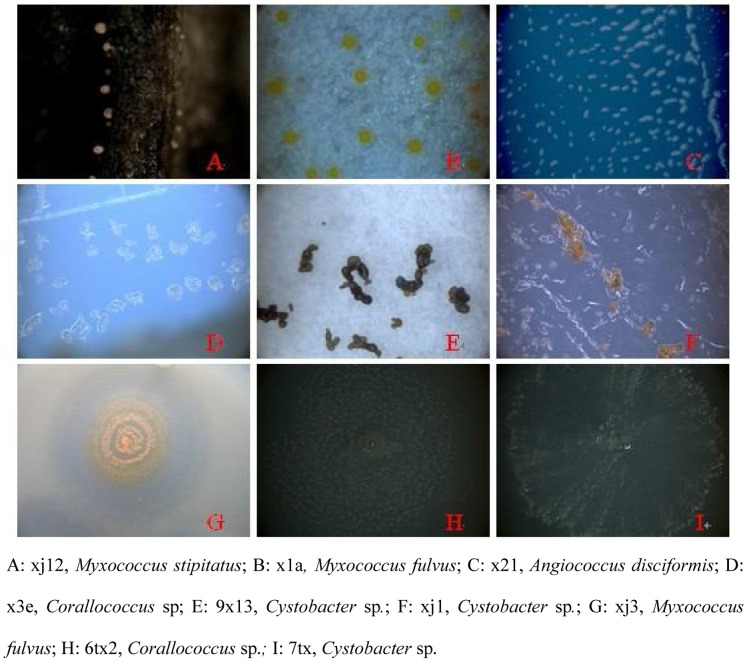
Morphological characteristics of myxobacterial fruiting bodies.

**Figure 2:**
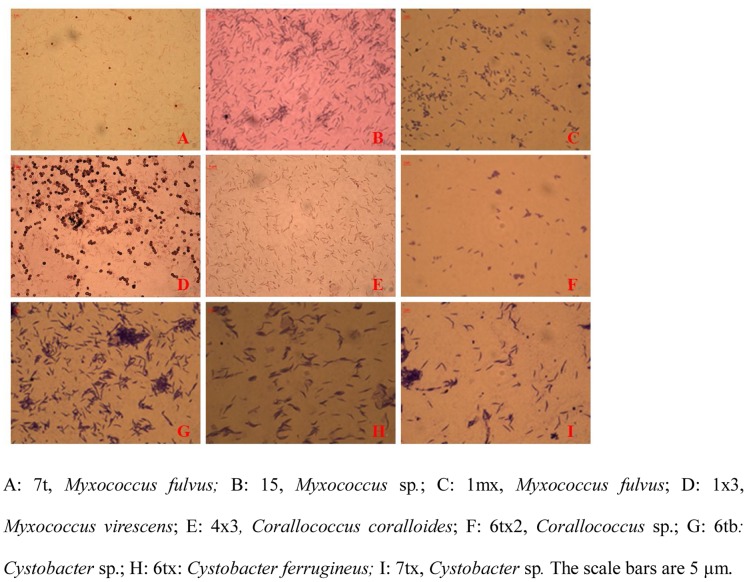
Optical micrograph of swarms of myxobacterial isolates (1000×).

**Figure 3:**
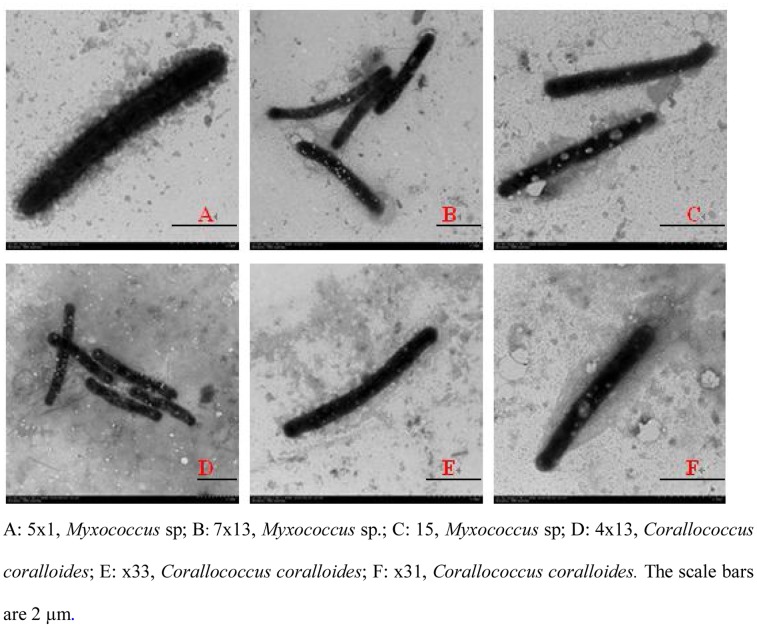
Electron micrograph of vegetative cells of new myxobacterial isolates (10,000×).

All of these strains were inoculated on VY/2 medium. Some myxobacterial fruiting bodies, swarms, and vegetative cells appeared (Fig. 1, 2, and 3, respectively). According to the main characteristics of myxobacteria previously described [1], [17], these strains were assigned to six genera: *Myxococcus* (33 strains), *Corallococcus* (14 strains), *Cystobacter* (6 strains), *Sorangium* (2 strains), *Nannocystis* (2 strains), and *Polyangium* (1 strain). *Myxococcus* strains were widely distributed in the soil environment, and tolerant to stresses, hence they could almost be isolated from every soil sample in the study. As described in Table 3, for *Myxococcus*, the fruiting bodies were always spherical, pale yellow, yellow, or orange red. Vegetative cells were always rod-shaped with sharp ends, 2–10 µm in length. For *Corallcoccus*, the fruiting bodies were always coralloid branched in shape; brown or orange red. Vegetative cells were rod-shaped with sharp ends, between 3–7 µm in length. In *Cystobacter*, the fruiting bodies always were ovoid and brown. Vegetative cells were rod-shaped with sharp ends and between 3–15 µm in length. Radial patterns were recognized within the swarm area.

**Table 3:** Morphological characteristics of the isolated myxobacteria.

Genera	Fruiting body in substrate	Fruiting body in VY/2	Vegetative Cell	Myxospore	Colony
*Myxococcus*	Spherical, large and solitary,wrapped by mucus, nacarat,pale yellow, yellow	Spherical, solitary, visible,yellow, nacarat, brown	slender, taperingends, 2–10 µm in length	Spherical, 1–2 µm	Large, spread swarm, yellow, nacarat,
*Cystobacter*	Assembled, large,ovoid, brown,	Without stipe, chainedor assembled, ovoid,brown	Slender rod-shape,tapering ends,3–15 µm in length	Thick rod-shape, 1–4 µm	spread swarm, distinct radial line, long edge with fruiting body, thin center
*Corallococcus*	Small, coralloid branch,nacarat, brown	In cluster, small,without stipe,nacarat	Slender, tapering ends,3–7 µm in length	Spherical, 1.0–2.5 µm in diameter	spread swarm, visible colony, easy to be picked
*Nannocystis*	Small, solitary, in cluster,covered by matrix, ovoid,	Sunk into medium,indistinct fruiting bodystructure	Short rod-shape,2.5–5 µm in length	Spherical or ovoid,0.8–1.2 µm in diameter	Agar corroded, distinct cyclic cristae, sunk into agar
*Polyangium*	Agar corroded, with radialline and spread swarm	Spherical, clump orsolitary, orange, yellow,light brown	Short rod-shape, bluntand round ends,2.5–7 µm in length	Similar morphologywith vegetative cells,2–5 µm in length	Agar corroded, with radial line and spread swarm
*Sorangium*	Grown in filter paperin cluster, colourless,yellow, brown	Small, sunk into medium,solitary, colourless	Short rod-shape bluntand round ends,2–10 µm in length	Similar morphologywith vegetative cells,1–3 µm in length	Agar corroded, fan-like spread swarm, fruiting body grown in the bottom of agar

### 16S r DNA Sequence Studies

The 16S rRNA gene sequences of 58 strains were compared by BLAST analysis (http://blast.ncbi.nlm.nih.gov/Blast.cgi) and EzTaxon(www.eztaxon.org), together with the morphologies of their vegetative cells, fruiting bodies, myxospores, and swarms, in order to determine their genera or specie. Among the 58 strains myxobacteria, 26 strains were identified to the species level, with the similarity of 99%–100%, while 31 strains were assigned to the genera level (Table 4). The level of similarity of xj4 and *Polyangium* sp. was 96%; 10×3 and x3t8 showed a similarity of 96% with *Sorangium cellulosum* and *Phaselicystis flava*.

**Table 4:** Identities of new myxobacterial isolates based on 16S r DNA analysis.

Assigned genera	Similarity (%)	Number of new isolates
*Myxococcus* sp.	99–100	22
*Myxococcus virescens*	100	1
*Angiococcus disciformis*	100	4
*Myxococcus fulvus*	100	5
*Cystobacter* sp.	96–99	6
*Corallococcus coralloides*	99–100	13
*Sorangium cellulosum, Phaselicystis flava*	96	2
*Nannocystis* sp.	99–100	2
*Myxococcus stipitatus*	100	1
*Polyangium* sp.	96	1
*Corallococcus* sp.	100	1

**Figure 4:**
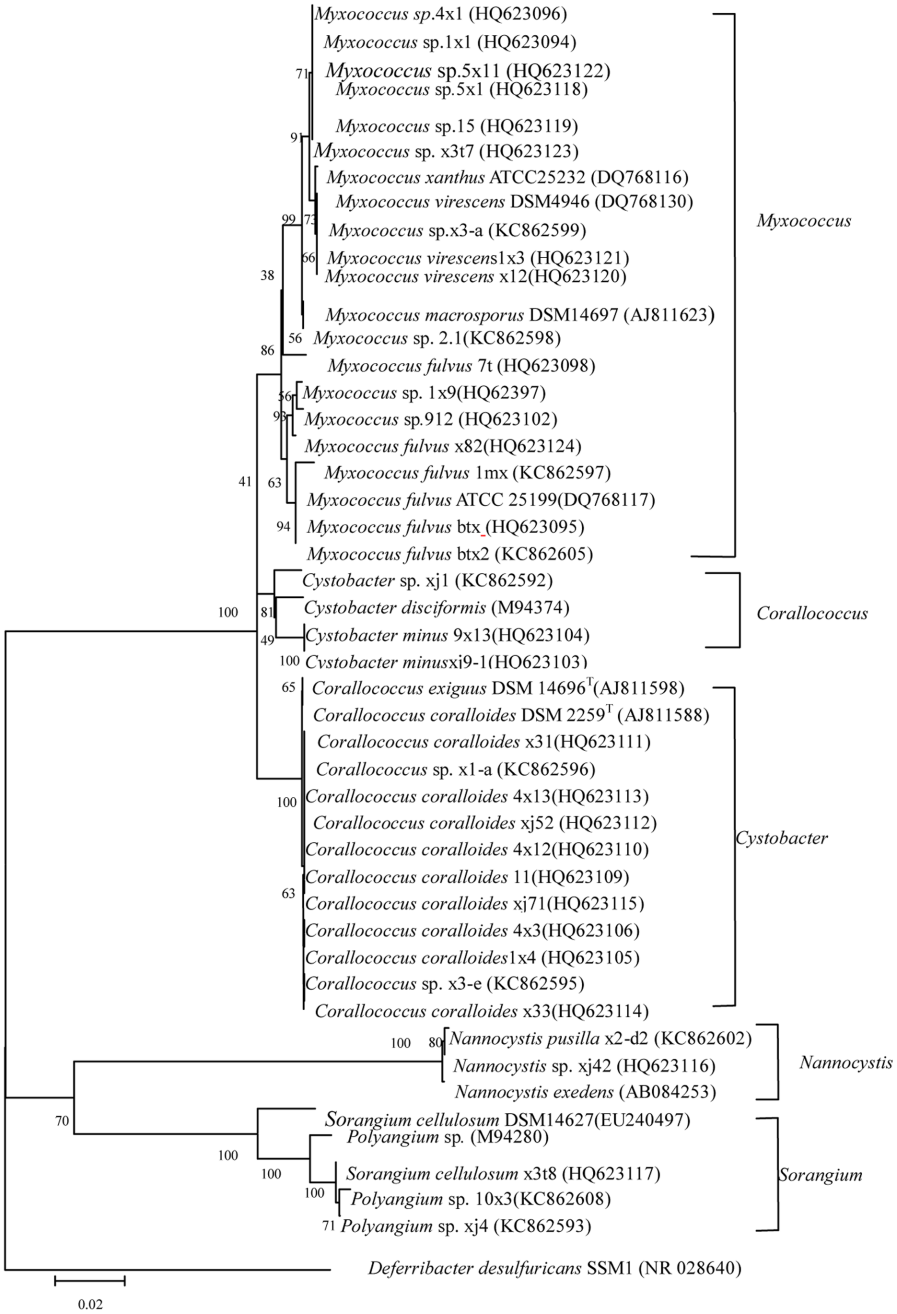
Phylogenetic tree of 36 new myxobacterial strains based on 16S rRNA gene sequences.

A phylogenetic analysis of 16S rDNA sequences from the 26 new isolates identified at the species level is shown in Fig. 4. These 26 strains were grouped into 5 branches: *Myxococcus*, *Corallococcus*, *Cystobacter*, *Sorangium*, and *Nannocystis.* For the *Myxococcus* branch, the level of similarity among 11 strains was 96%. For *Corallococcus*, the closest relatives among the 10 strains were *Corallococcus coralloides* and *Corallococcus exiguou*s (level of similarity 100%). The closest relative of 9×13 and xi9-1 was *Cystobacter disciformis* (level of similarity 91%). The closest relative of xj42 was *Nannocystis exedens* (level of similarity 100%). The level of similarity of x3t8, xj103, and *Sorangium cellulosum* was also 100%.

### Taxonomic Characteristics

#### Physiology and biochemical characteristics of new strains

The physiological features of eleven new isolates are shown in the Table 5. All 11 strains were negative for oxidase and positive for catalase, and hydrolyzed casein, starch, and Tween 80. Almost all *Myxococcus* and *Croallcoccus* strains grew within a temperature range of 10–37°C, with the exception of 7t, which did not grow at 10°C. *Cystobacter* sp. strain xj9-1 grew within a temperature range of 20–37°C. The nitrate reduction reaction was negative for all *Myxococcus* and *Croallcoccus* strains, while positive for *Myxococcus* sp. and *Croallcoccus* sp.

**Table 5:** Physiological properties of new isolates from saline-alkaline soils.

Strain physiological test	*Myxococcus*	*Croallcoccus*	*Cystobacter*
	8.5, 7t, 1×3, 912, x3t7	xj71, 4×3, xj52, x33, x31	xj9-1
Hydrolysis Tween 80	+	+	+
Catalase	−	−	−
Oxidase	+	+	+
Growth at: 10°C	+(7t−)	+	−
20°C	+	+	+
30°C	+	+	+
37°C	+	+	+
45°C	− (7t +)	−	+
Nitrate reduction	−	−	+
Starch (0.2%, 1%) hydrolysis, halo	+	+	+

#### Fatty acid analysis

Analyses of fatty acids were performed on several representatives of each of the genera and some reference strains (*Corallococcus macrosporus* DSM 14697, *Corallococcus coralloides* DSM 52499, DSM 16525, *Myxococcus stipitatus* DSM14675, DSM 2260, *Cystobacter violaceus* DSM14727) (Table 6). All *Myxococcus* sp. and *Corallococcus* sp. reference strains contained substantial quantities of C15∶0iso branched fatty acids. Many also contained C17∶0iso branched fatty acids. In *Cystobacter*, the reference strains contained the highest quantities of C16∶1 ω5c branched fatty acids and also many C15∶0iso branched fatty acids.

**Table 6:** Major fatty acids of new isolates and reference strains.

Fatty acid	xj71^a^	DSM14697	DSM52499	1×3^a^	DSM16525	DSM14675	DSM2260	xj9-1^a^	DSM14727
C14∶0	0.6	1.6	0.7	0.6	1.6	0.8	1.1	2.0	1.6
C16∶0	0.9	2.4	3.0	1.4	4.2	3.9	3.0	5.2	2.8
C13∶0iso	3.7	0.8	3.0	0.5		0.5	0.5	0.2	0.2
C14∶0-3OHiso	7.0	7.4	3.6	18.0	9.5	12.7	6.6	0.2	11.1
C15∶0iso	36.4	41.9	27.6	41.7	29.2	33.2	35.7	10.5	19.8
C16∶0iso	2.3	0.9	2.3	0.7		1.0	0.4	5.4	12.8
C17∶0iso	18.9	11.3	20.0	6.9	8.6	8.0	13.2	5.1	5.0
C17: anteiso	12.3	0.1	10.9		0.6	5.1	0.4	0.2	0.5
C17:-3OHiso	1.8	3.4	4.8	3.6	4.3	5.3	2.3	1.3	4.2
C17∶1ω10ciso	2.5	1.5	1.4		1.8	0.7	1.3		
C16∶1 ω5c		8.8		6.3	9.3	13.6	7.7	31.4	22.3
C16∶1 w7c	5.4	6.7	5.8	5.5	7.7	3.6	7.4	13.6	1.8
C18∶1 w7c								12.7	

a New isolates.

#### DNA base composition

Five of the new isolates were selected to analyze G+Cmol% (Table 7). The G+C content of these isolates ranged from 70.0–73.4%.

**Table 7:** G+Cmol% of some test strains.

Strain	Genera	G+Cmol%
1×3	*Myxococcus virescens*	71.80
btx2	*Myxococcus fulvus*	72.02
xj71	*Corallococcus coralloides*	73.39
xj9-1	*Cystobacter* sp.	72.57
xj42	*Nannocystis* sp.	70.19

#### MLSA analysis

**Figure 5:**
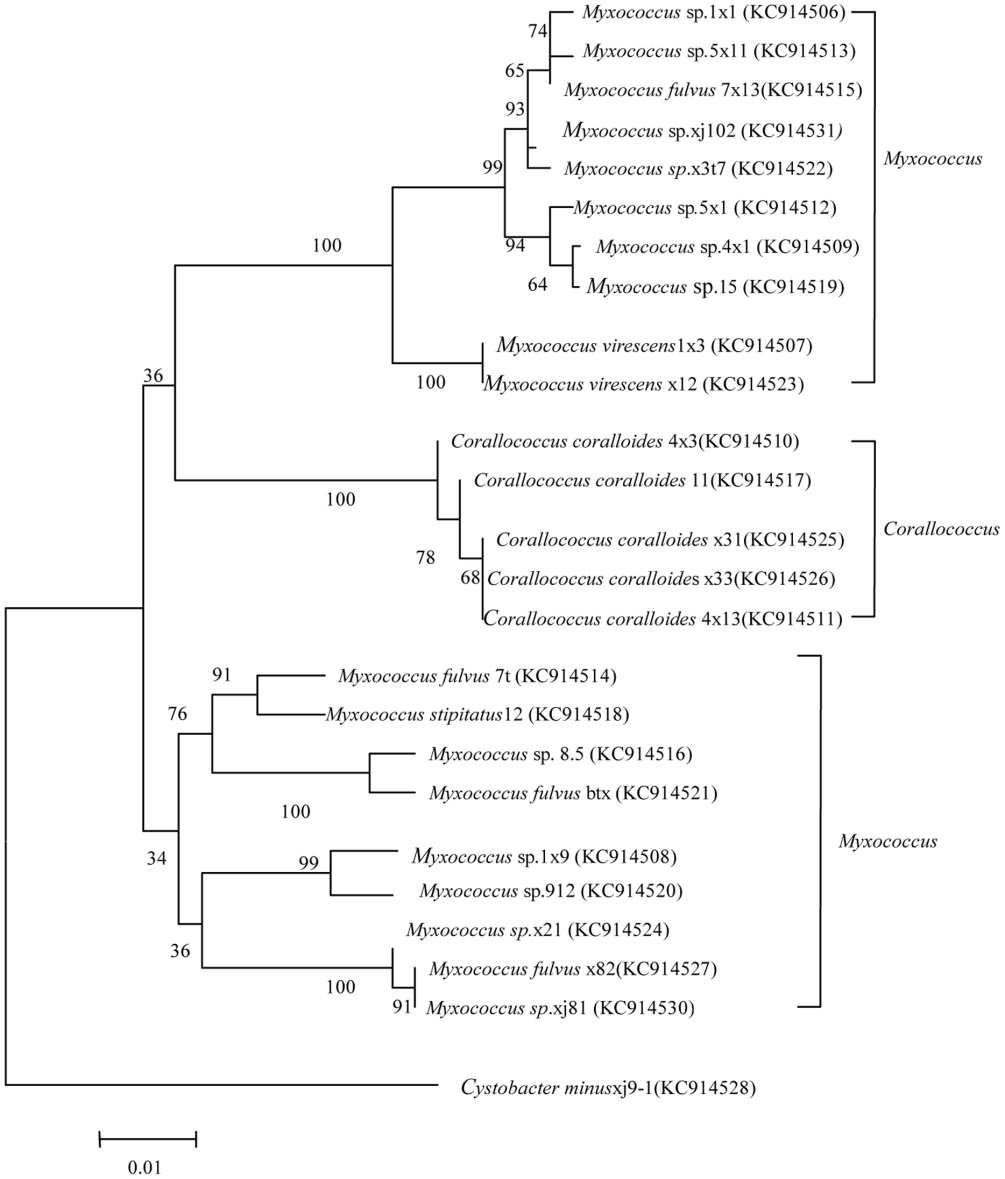
Phylogenetic relationships among 25 myxobacterial strains, inferred from *lepA* sequences.

**Figure 6:**
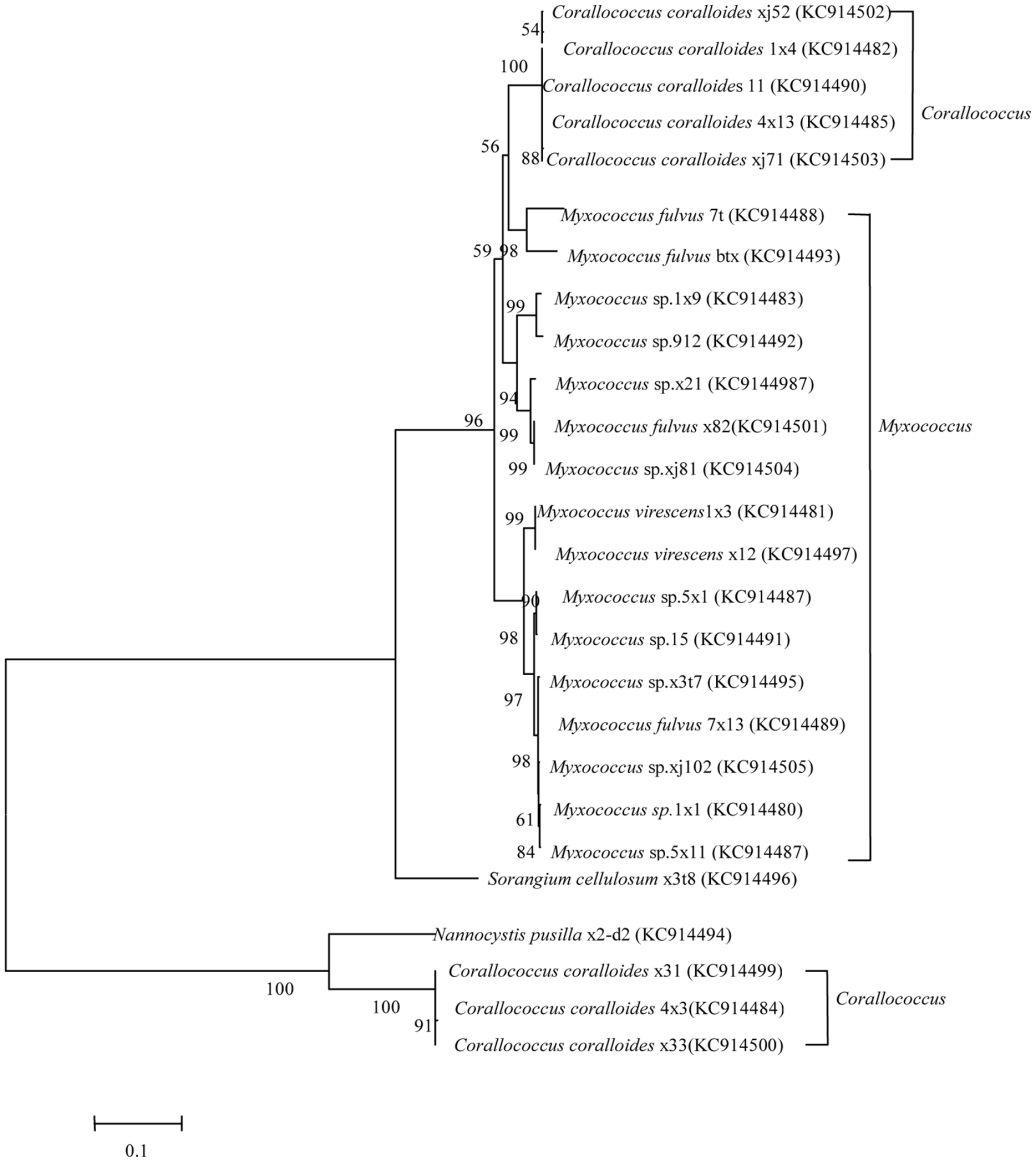
Phylogenetic relationships among 26 myxobacteria strains, inferred from *gyrB* sequences.

The two protein-coding genes were selected for MLSA studies by Santos and Ochman [25]. Note that although the primers used are considered universal, they were not successful for all of our new strains. For example, using the *lepA* primer, *lepA* could not be amplified from 7t, x82, xj81, 7×13, 1×1, or x3t8. For *lepA* and *gyrB*, the topologies of gene trees with neighbor-joining analyses were similar, though not identical (Figs. 5 and 6, respectively).

### Effects of NaCl on Growth of Myxobacteria

As shown in Table 8, the growth of the reference terrestrial strains tested was inhibited by high salinity. All tested strains were found to grow at 1% NaCl (w/v). Only some *Myxococcus* strains and one *Cystobacter* sp. strain were able to grow at 2% NaCl (w/v). No reference strains grew at NaCl concentrations over 2% (w/v).

**Table 8:** Growth of some of myxobacteria at different NaCl concentrations.

Name	Genera or species	Salinity concentration				
		1%	2%	3%	4%	5%
4×2	*Myxococcus* sp.	+++++	++	−	−	−
x5	*Myxococcus* sp.	+++	+	−	−	−
x3t7	*Myxococcus* sp.	+++	−	−	−	−
1×9	*Myxococcus* sp.	+++	−	−	−	−
1×4	*Corallococcus coralloides*	+++	+	−	−	−
1×3	*Myxococcus* sp.	+++++	−	−	−	−
8.5	*Myxococcus fulvus*	+++	−	−	−	−
7t	*Myxococcus fulvus*	+++	−	−	−	−
xj52	*Corallococcus coralloides*	+++++	−	−	−	−
xj71	*Corallococcus coralloides*	+++	−	−	−	−
x31	*Corallococcus coralloides*	+++++	−	−	−	−
912	*Myxococcus* sp.	+++++	+	−	−	−
4×3	*Corallococcus coralloides*	+++++	−	−	−	−
5×1	*Myxococcus* sp.	+++++	+++	−	−	−
15	*Myxococcus* sp.	+++++	++	−	−	−
1×1	*Myxococcus* sp.	+++++	+	−	−	−
9×13	*Cystobacter* sp.	+++++	+	−	−	−

“+” (1–5) represents growth. “−” indicates no growth.

## Discussion

Fifty-eight myxobacterial strains were isolated from saline-alkaline soils in Xinjiang, China. Thirty-three strains were found to belong to the genus *Myxococcus*. There were 14 *Cystobacter* spp., 6 *Corallococcus* spp., 2 *Sorangium* spp., 2 *Nannocystis* spp., and 1 *Polyangium* sp.

The taxonomy of myxobacteria is based mainly on the characteristics of the fruiting bodies. However, fruiting bodies are often lost during purification, so it is difficult to identify species such as *Myxococcus* sp. and *Corallococcus* sp. Furthermore, the comparison of 16S rRNA gene sequences has been shown to be only minimally useful for discriminating closely related strains [27]. MLSA, however, is a promising method for bacterial systematics [26]. Here we used both 16S rRNA gene analysis and MLSA to identify myxobacteria. As shown in Fig. 4 and Fig. 6, the 16S rRNA gene sequence analysis was found to facilitate the division of samples into genera. For species, however, MLSA was found to be the superior method.

Usually terrestrial myxobacteria cannot grow at NaCl concentration higher than 1%. Our work showed that 7 *Myxococcus* sp. were capable of growing at NaCl concentrations of 2%. They were also found to form fruiting bodies more quickly at NaCl concentrations of 1% than in the absence of NaCl. We therefore concluded that these 7 isolates were specifically adapted to saline-alkaline environments.

This is the first report describing the phylogenetic and physiological characteristics of myxobacteria isolated from saline-alkaline ecosystems. These results provide us with new information about the ecology of myxobacteria, and the methodologies provided here will be useful for further studies of the ecology of these interesting and economically important organisms.

It is clear that the diversity of myxobacteria is low in saline-alkaline soils in Xinjiang. This can be explained by the following reasons. First, the number of soil microbes in saline-alkaline soil is limited. The extreme physio-chemical property and condition in the saline-alkaline environment strongly affect the diversity of microbes, resulting in an unfavorable growth of microbes. However, it is the special characteristics of the saline-alkaline environment that may support much more new unidentified species. Second, the isolation methods are traditional. The conventional rabbit dung pellets method, WCX method, direct heat method, antibiotic mixture method and filter paper method have their own limitations, and they are specific to some myxobacteria but not to all of them. For instance, rabbit dung pellets method is usually used for isolating *Myxococus fulvus, Corallococcus coralloides, Cystobacter fuscus, Cystobacter ferrugineus, Archangium gephyra, Stigmatella erecta, Myxococcus vtrescens, Myxococus xanthus* and *Cystobacter velatus* [28], [29]; whereas filter paper method is commonly specified to *Stigmatella aurantiaca*, *Chondromyce apiculatus*, *Corallococcus coralloides*, *Myxococus.fulvus*, *Chondromyce pedicularus*, *Haploangium* sp., *Chondromyces* sp. and *Stigmatella* sp. [30]. The limitation of isolation methods causes the loss of myxobacteria discovery. Third, the salinity is high in the saline-alkaline soil, which is not suitable for the growth of myxobacteria. Myxobacteria are prone to the habitat rich in organic matter, with pH 6–8. It has been documented that terrestrial myxobacteria can not grow above 1% salinity [1]. The pH in the saline-alkaline soils of Xinjiang ranges from 7.6 to 8.5, and the salinity of soil is high, ultimately the species are scarce. Last, myxobacteria are also affected by soil property. Myxobacteria are abundant in soil; brown soil and black soil are rich in nutrient, and myxobacteria. But sandy soil and decomposed rock soil are lacking myxobacteria [31]. The sandy soil in Xinjiang is not suitable for the growth of myxobacteria.

In the evolution of 16S rRNA sequences, strains 1×1, 15, 5×1, 5×11, and x3t7 clustered together, with the similarity of 66%. While in the phylogenetic relationship of *gyrB,* a close cluster is observed. Among them, 15 and 5×1 clustered with a similarity of 98%; 1×1 and 5×1 clustered with a similarity of 98%; 7×13 and x3t7 clustered with a similarity of 84%. Hence, phylogenetic relationship of *gyrB* may be more suitable to reveal the phylogenetic relationship of those strains.
